# Immune Response Generated With the Administration of Autologous Dendritic Cells Pulsed With an Allogenic Tumoral Cell-Lines Lysate in Patients With Newly Diagnosed Diffuse Intrinsic Pontine Glioma

**DOI:** 10.3389/fonc.2018.00127

**Published:** 2018-04-26

**Authors:** Daniel Benitez-Ribas, Raquel Cabezón, Georgina Flórez-Grau, Mari Carmen Molero, Patricia Puerta, Antonio Guillen, E. Azucena González-Navarro, Sonia Paco, Angel M. Carcaboso, Vicente Santa-Maria Lopez, Ofelia Cruz, Carmen de Torres, Noelia Salvador, Manel Juan, Jaume Mora, Andres Morales La Madrid

**Affiliations:** ^1^Department of Immunology, Hospital Clinic, August Pi i Sunyer Biomedical Research Institute (IDIBAPS), University of Barcelona, Barcelona, Spain; ^2^Department of Clinical Trials, Hospital Sant Joan de Déu, Barcelona, Spain; ^3^Department of Neurosurgery, Hospital Sant Joan de Déu, Barcelona, Spain; ^4^Laboratory of Developmental Cancer, Institut de Recerca Sant Joan de Déu, Barcelona, Spain; ^5^Department of Oncology and Hematology, Hospital Sant Joan de Déu, Barcelona, Spain; ^6^Department of Neuro-Oncology, Hospital Sant Joan de Déu, Barcelona, Spain; ^7^Department of Immunotherapy, Hospital Sant Joan de Déu, Barcelona, Spain

**Keywords:** immunotherapy, dendritic, cell, vaccination, diffuse intrinsic pontine glioma

## Abstract

**Background and objective:**

Diffuse intrinsic pontine glioma (DIPG) is a lethal brainstem tumor in children. Dendritic cells (DCs) have T-cell stimulatory capacity and, therefore, potential antitumor activity for disease control. DCs vaccines have been shown to reactivate tumor-specific T cells in both clinical and preclinical settings. We designed a phase Ib immunotherapy (IT) clinical trial with the use of autologous dendritic cells (ADCs) pulsed with an allogeneic tumors cell-lines lysate in patients with newly diagnosed DIPG after irradiation (radiation therapy).

**Methods:**

Nine patients with newly diagnosed DIPG met enrollment criteria. Autologous dendritic cell vaccines (ADCV) were prepared from monocytes obtained by leukapheresis. Five ADCV doses were administered intradermally during induction phase. In the absence of tumor progression, patients received three boosts of tumor lysate every 3 months during the maintenance phase.

**Results:**

Vaccine fabrication was feasible in all patients included in the study. Non-specific KLH (9/9 patients) and specific (8/9 patients) antitumor response was identified by immunologic studies in peripheral blood mononuclear cells (PBMC). Immunological responses were also confirmed in the T lymphocytes isolated from the cerebrospinal fluid (CSF) of two patients. Vaccine administration resulted safe in all patients treated with this schema.

**Conclusion:**

These preliminary results demonstrate that ADCV preparation is feasible, safe, and generate a DIPG-specific immune response detected in PBMC and CSF. This strategy shows a promising backbone for future schemas of combination IT.

## Background

Diffuse intrinsic pontine glioma (DIPG) accounts for the vast majority of brainstem tumors in children. Upfront focal irradiation is the only effective palliative therapy, achieving clinical and radiologic response in the majority of patients. Unfortunately, irreversible tumor re-growth is the rule during the following 2 years after initial presentation (1). DIPG molecular characterization has unveiled specific alterations and key biologic pathways showing that we are not in front of just one entity, but of a heterogeneous group of tumors that share clinical and radiologic characteristics and similar poor prognosis. These molecular alterations are now starting to be translated into targeted therapies (2–5). Also, new delivery systems that attempt to circumvent the blood–brain barrier are being tested (6) and exciting preclinical and clinical projects—*including IT*—may change soon our therapeutic approach to this infiltrative glioma (7–12). Immunotherapy (IT) is gaining popularity as a potential efficacious therapeutic option for central nervous system (CNS) aggressive glial tumors both in adults and children (13). Several decades ago, different groups reported that gliomas promote both local and systemic immunosuppression to a certain degree. Numerous cell-based pathways of immunosuppression were first identified in glioblastoma multiforme, and these observations collectively support the hypothesis that immune system modulation could have therapeutic potential. The CNS, historically considered as immune privileged and “protected” site, is a dynamic immunological environment in which astrocytes, microglia, and a variety of infiltrating immune cells play a major role in these structures during host immunity to antigens, representing a valuable weapon to fight effectively cancer maintenance and progression (13, 14). Importantly, these strategies may be of particular relevance for non-resectable, radio-resistant, invasive, migratory, and aggressive gliomas. Even though DIPG’s “epicenter” is identified radiologically at diagnosis in the pons, is now well recognized that early or late microscopic and/or macroscopic dissemination plays a key role in tumor progression and dismal outcome (15). Consequently, the future potential effective therapeutic schema for DIPG will surely need a mechanism that secures “whole CNS control,” since we need to address its dissemination capacity from diagnosis, even in the absence of macroscopic disease by imaging.

Currently, two different IT strategies are being tested specifically for DIPG in early clinical trials (clinicaltrials.gov: NCT02960230 and NCT02359565). There is enough accumulated data that support the idea that Autologous dendritic cell vaccine (ADCV) can satisfactory induce maintained antitumor immunological responses. These IT approach has achieved encouraging clinical and radiologic results in cases with advanced cancers (16–19). We expect that dendritic cells (DCs) loaded with a variety of DIPG tumor antigens, will have the ability to induce antitumor immune responses in patients with newly diagnosed DIPG after upfront irradiation. Based on our accessibility to robust molecularly tested DIPG cell lines immortalized in our laboratory, we decided to explore the potential use of autologous dendritic cells (ADCs) pulsed with an allogeneic tumor cell-lines lysate (ATCL) rich in tumor antigens and potentially neo-antigens in patients with newly diagnosed DIPG after upfront irradiation. The principal objective of this pilot study was to establish the feasibility of preparation and safety of intradermic administration of ADCVs in patients with newly diagnosed DIPG after radiation therapy (RT). Additionally, to evaluate the non-specific and specific antitumoral immune response generated.

## Patients and Methods

### Study Design

This is an institutional phase Ib trial at Hospital Sant Joan de Déu, in collaboration with Hospital Clínic, both in Barcelona, Spain. The ethics committee at both institutions and the Spanish regulatory Agency for novel products and medications approved the clinical trial prior patient accrual (AEMPS; PEI 15-215, and PEI 15-216, clinicaltrials.gov: NCT02840123). Parents or legal guardians provided written informed consent at patient inclusion. Eligible patients included cases with newly diagnosed non-metastatic DIPG based upon radiologic criteria which showed clinical and radiologic response to upfront RT. Tumor biopsy was not mandatory for trial inclusion. A Lansky performance of 50 or above, life expectancy of more than 8 weeks and basic hematologic and metabolic parameters within normal limits were mandatory. Exclusion criteria included tumor progression after RT, metastatic disease at diagnosis or after RT and steroids dependency for neurologic stability. Other exclusion criteria included active or uncontrolled infections and concurrent immunosuppressive treatment or infection by HIV, HBV, or HCV.

Patient’s DCs were derived from monocytes obtained by leukapheresis at diagnosis or at completion of RT once steroids had been discontinued. These monocytes-derived DCs were loaded with an ATCL composed of eight cell lines obtained and matured *ex vivo* from five patients treated in the past in our institution with H3K27 mutated DIPG. Patients included in the trial were planned to receive five intradermal injections (0.4 ml/injection) containing 10 × 10^6^ mature ADCs previously pulsed with the above mentioned tumor lysate. The first dose administration was planned 3–6 weeks after the completion of RT. Following doses were planned in an every other week basis. Three months after the last dose of the induction phase and in the absence of clinical or radiologic progression, patients re-started therapy with boosts of DIPG ATCL as a maintenance phase for a total of three doses.

### Tumor Samples and Preparation of ATCL

#### Tumor Samples

Primary tumor-sphere cultures from five DIPG patients named after the codes HSJD-DIPG-007, HSJD-DIPG-008, HSJD-DIPG-012, HSJD-DIPG-013, and HSJD-DIPG-014 were established from fresh tumor fragments. In brief, tumor tissues were disaggregated using a mixture of collagenase (5 mg/mL) and DNAse (400 Kunitz Units/mL) from Sigma (St. Louis, MO, USA) at 37°C during 5 min to obtain single cell suspensions. Cells were cultured in a mixture of Dulbecco’s modified Eagle’s medium/F-12 and neurobasal-A medium at a 1:1 ratio with HEPEs, sodium pyruvate, non-essential aminoacids solution, glutamax, and antibiotic-antimycotic supplemented with 2% B27 (all from Life Technologies, Grand Island, NY, USA), and growth factors EGF (20 ng/mL), bFGF (20 ng/mL), PDGF-AA, and PDGF-BB (10 ng/mL), all from Peprotech (Rocky Hill, NJ, USA) and 2 µg/mL heparin (Sigma). Additionally, cells HSJD-DIPG-007, HSJD-DIPG-012, and HSJD-DIPG-013 were cultured in the absence of supplements and growth factors and adding 10% fetal bovine serum to establish adherent cultures. Thus, five tumor-sphere cultures and three adherent cultures were used to produce the final eight ATCL.

#### Preparation of ATCL

Diffuse intrinsic pontine glioma cell lines were pooled together and 50% of these pooled cells were treated with dinitrofluorobenzene (DNFB) for 15 min at room temperature to increase immunogenicity. After extensive washing with phosphate-buffered saline (PBS), pooled and DNFB treated cells were successively incubated for five cycles of 1 min in liquid nitrogen and at 37°C freezing and thawing to produce the cell lysate. Aliquots of 1 mL of this 10 × 10^6^ cells/mL of PBS were irradiated at 25 kGy in order to sterilize the product. The absence of viral, bacterial, or any cell growth was confirmed in the final product.

### Preparation of ADCVs

After inclusion in the study, leukapheresis was performed to obtain peripheral blood leukocytes. Autologous monocytes were selected by adherence to plastic, and then differentiated to DCs for 7 days in synthetic X-VIVO 15 media (Lonza, Walkersville, MD, USA) supplemented with 2% autologous serum and differentiation cytokine mix [800 U/ml granulocyte-macrophage colonies stimulating factor (Miltenyi Biotech, Bergisch Gladbach, Germany) + 500 U/ml IL-4 (Miltenyi Biotec, Bergisch Gladbach, Germany)]; an additional 24 h in a maturation cytokine mix [10 ng/ml TNF-α (Miltenyi Biotech, Bergisch Gladbach, Germany) + 10 ng/ml IL1-β (Miltenyi Biotech, Bergisch Gladbach, Germany) + 10 ng/ml IL-6 (Miltenyi Biotech, Bergisch Gladbach, Germany) + 1 µg/ml prostaglandin E2 (PGE2; Dinoprostona, Pfizer, New York, NY, USA) + 20 µg/ml poly(I:C) (Hiltonol^®^; Oncovir Inc, Washington DC, USA) (courtesy of Dr. Andres Salazar)] in the presence of ATCL plus KLH 10 µg/ml (IMMUCOTHEL^®^ from Biosyn) using good manufacturing practice standard procedures. DC maturation was confirmed by assessing increases in the immunofluorescence of CD80, CD83, and CD86 (all from BD Bioscience, San Diego, CA, USA) and HLA-DR (BD Bioscience, San Diego, CA, USA), and purity determined by the absence of CD3 (T lymphocytes) (BD Bioscience, San Diego, CA, USA), CD14 (Monocytes) (BD Bioscience, San Diego, CA, USA), and CD19 (B lymphocytes) (BD Bioscience, San Diego, CA, USA). Flow cytometry analysis was performed using FACS Canto II and FACSDIVA^®^ software (BD Bioscience, San Diego, CA, USA). Release criteria included >80% CD80+, CD86+ and negative microbial test (sterility and mycoplasma detection) according to European Pharmacopeia.

### Immunological Monitoring

Peripheral blood mononuclear cell (PBMC) and serum were collected every 2 weeks, starting baseline and prior to each vaccine dose during the induction phase. PBMCs were labeled with 5,6-carboxyfluorescein diacetate succinimidyl ester (CFSE) to evaluate cell division. To determine the effect of ADCVs in the non-specific and specific antitumor immune response, the presence of tumor lysate specific T-cells was evaluated, before and after treatment, incubating PBMC with KLH or ATCL, respectively. Plates were incubated in a humid atmosphere of 5% CO_2_ at 37°C for 7 days. T-cell proliferation (cell dilution) was evaluated by a flow cytometry-based assay and quantified as the percentage of proliferating (CFSE-diluted) cells. When present, T-cells were expanded from cerebrospinal fluid cerebrospinal fluid (CSF) (taken prior to first dose of vaccine, 2 weeks after dose 4 and dose 5) by using anti-CD3-CD28 beads and rhIL-2. Expanded T-cells were incubated with autologous mature DCs loaded with KLH or tumor cell line lysate with the same goal as described above. Cells were incubated in a humid atmosphere of 5% CO_2_ at 37°C for 7 days. Eighteen hours before termination of culture, each well received 0.5 μCi of methyl-3H-thymidine at 2 Ci/mmol (TRA310; Amersham Biosciences, Little Chalfont, UK). Uptake of thymidine into DNA was determined using a cell harvester (Perkin Elmer, Massachusets, USA). We calculated the geometric mean of three replicates.

## Results

### Patient Characteristics

To date, 20 patients were evaluated for eligibility for the trial. Eleven patients were not eligible. All of them were not eligible due to local or metastatic tumor progression at the end of RT. Nine patients between 4 and 10 years of age were enrolled in the trial. Demographic, clinical, and tumor tissue data are summarized in Table 1.

**Table 1:** Patients characteristics.

Patient ID	Age (years)	Sex	Biopsy	Histologic diagnosis	K27M status	Radiation therapy (dose)	Steroids discontinued prior to enrollment
DIPG-DC001	7	F	N	N/A	N/A	Y (54 Gy)	Y
DIPG-DC002	5	F	Y	K27M Midline diffuse glioma	K27M H3.3 mutated	Y (54 Gy)	Y
DIPG-DC003	7	F	Y	K27M Midline diffuse glioma	K27M H3.3 mutated	Y (54 Gy)	Y
DIPG-DC004	7	M	N	N/A	N/A	Y (54 Gy)	Y
DIPG-DC006	5	F	Y	High-grade glioma (HGG). IHC suggest WT K27M	N/A	Y (54 Gy)	Y
DIPG-DC007	5	F	N	N/A	N/A	Y (54 Gy)	Y
DIPG-DC008	10	F	Y	K27M Midline diffuse glioma	K27M H3.3 mutated	Y (54 Gy)	Y
DIPG-DC009	4	M	N	N/A	N/A	Y (39 Gy)—hypofractionated	Y
DIPG-DC010	4	F	Y	HGG	K27M WT	Y (54 Gy)	Y

*Y, yes, N, no, IHC, immunohistochemistry, WT, wild type*.

### ADCVs Preparation

All patients included in the trial went successfully through leukapheresis. No complications secondary to the procedure were reported. Enough mononuclear cells were recovered for the preparation of the five doses needed for the induction phase for all cases.

### Therapy Administered

All patients received upfront focal irradiation as part of DIPG standard of care. Clinical and radiologic response was confirmed prior to study entry and distant dissemination was ruled out by brain and spine MRI.

#### Induction Phase

In the absence of new clinical symptoms, patients received five outpatient ADCVs doses during this phase (AEMPS PEI 15-215). Forty-four dosages of ADCVs were administered. One patient did not receive the fifth dose of the induction phase due to tumor progression.

#### Maintenance Phase

**Figure 1:**
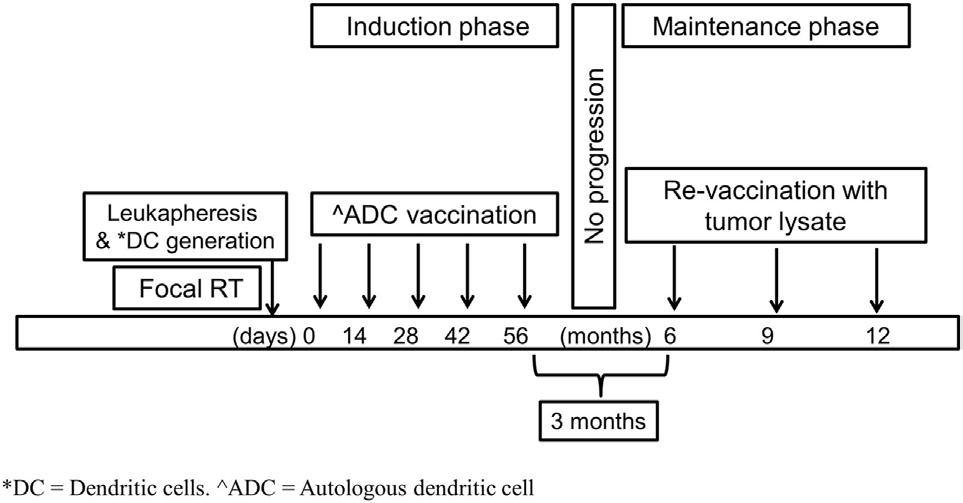
Therapeutic schema.

Three months after the last induction phase dose—in the absence of tumor progression—three boost dosages of ATCL (AEMPS PEI 15-216) 3 months apart were administered intradermically (Figure 1).

**Figure 2:**
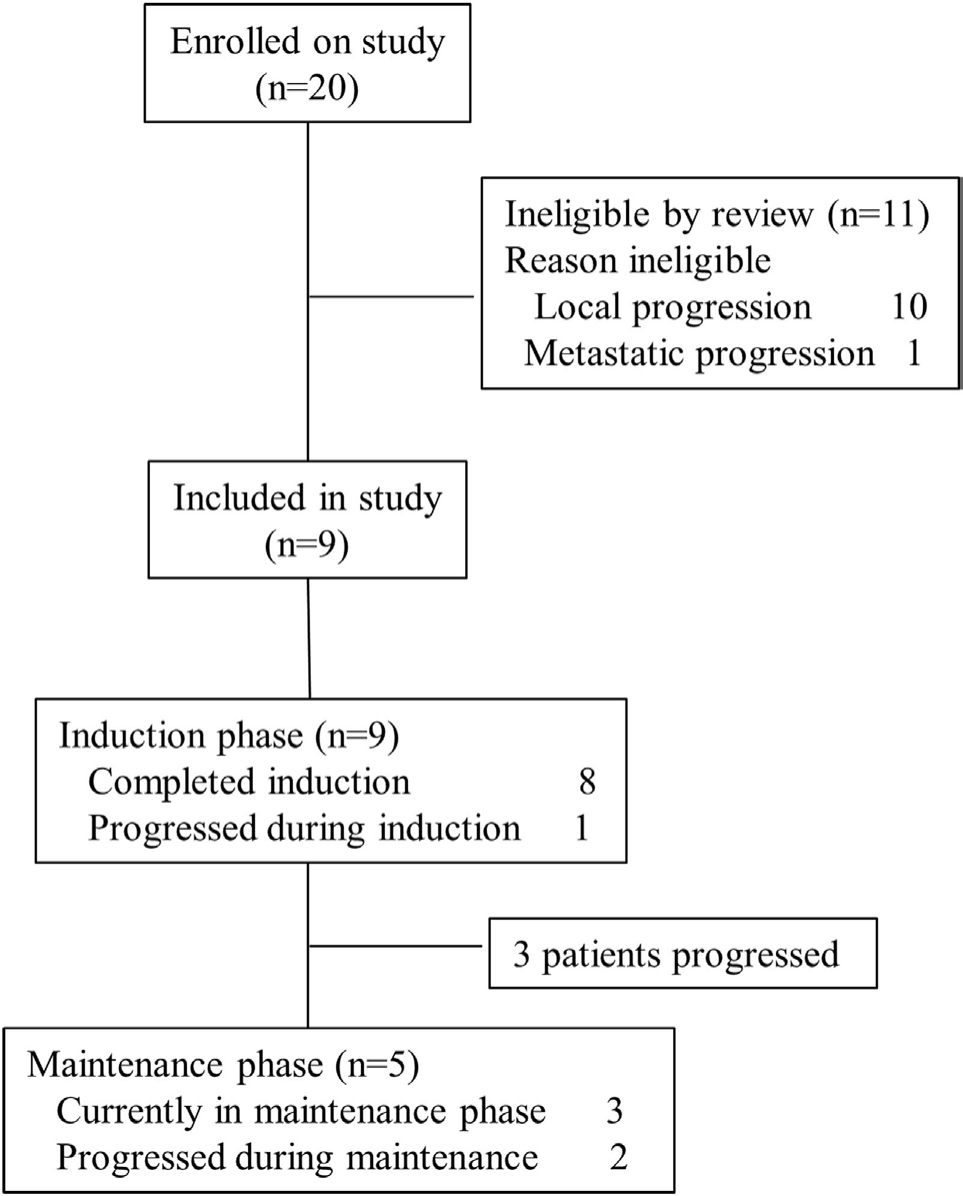
Consort diagram.

Up to the date of this report, four dosages of ATCL have been administered to three patients and three patients are still on trial within the maintenance phase (Figure 2).

### Toxicity

Intradermally administration of ADCVs was overall well tolerated. Therapy was administrated in an outpatient regimen with no significant adverse events (AEs) identified. Although mild local pain and/or mild itching were reported during intradermally dosages administration, no relevant systemic symptoms were reported after vaccination. Mild headaches and nausea were reported in a few dosages in most patients. Laboratory abnormalities were mild and no clinically relevant. Patient DIPG-DC006 developed an intratumoral hemorrhage after the second dose of the induction phase. Neurologic deterioration prompted delayed administration of doses 3 and 4. Patient DIPG-DC010 developed recurrent biopsy-tract infections: the first episode was recorded prior to the initiation of IT. When enrolled in the study, infection was under control. Thereafter, we do not think the infection is related to the ADCVs. Table 2 shows the list of AEs observed during therapy for all the patients enrolled.

**Table 2:** Adverse events (AEs).

Patient ID	AE	CTCAE grade (max. grade)
DIPG_DC001	Headaches	1
	Nausea	1
	Anemia	1

DIPG_DC002	Headaches	1
	Nausea	1
	Vomiting	1
	Anemia	1
	Leukopenia	1

DIPG_DC003	Headaches	2
	Nausea	2
	Vomiting	2
	Anemia	1
	Leukopenia	1

DIPG_DC004	Headaches	1
	Vomiting	1
	Leukopenia	1
	Thrombocytopenia	1

DIPG_DC006	Headaches	2
	Alopecia	1
	Anemia	1
	Leukopenia	1

DIPG_DC007	Nausea	1
	Vertigo	2
	Anemia	1
	Leukopenia	1

DIPG_DC008	Headaches	1
	Nausea	1
	Leukopenia	1
	Neutropenia	1
	Vomiting	1

DIPG_DC009	Headaches	1
	Vomiting	1
	Leukopenia	1

DIPG_DC010	Headaches	1
	Nausea	1
	Osteomielitis	3
	Anemia	1
	Leukopenia	1
	Lymphopenia	1
	Hypokalemia	1
	Hypertension	2

### Vaccines Characterization

**Figure 3:**
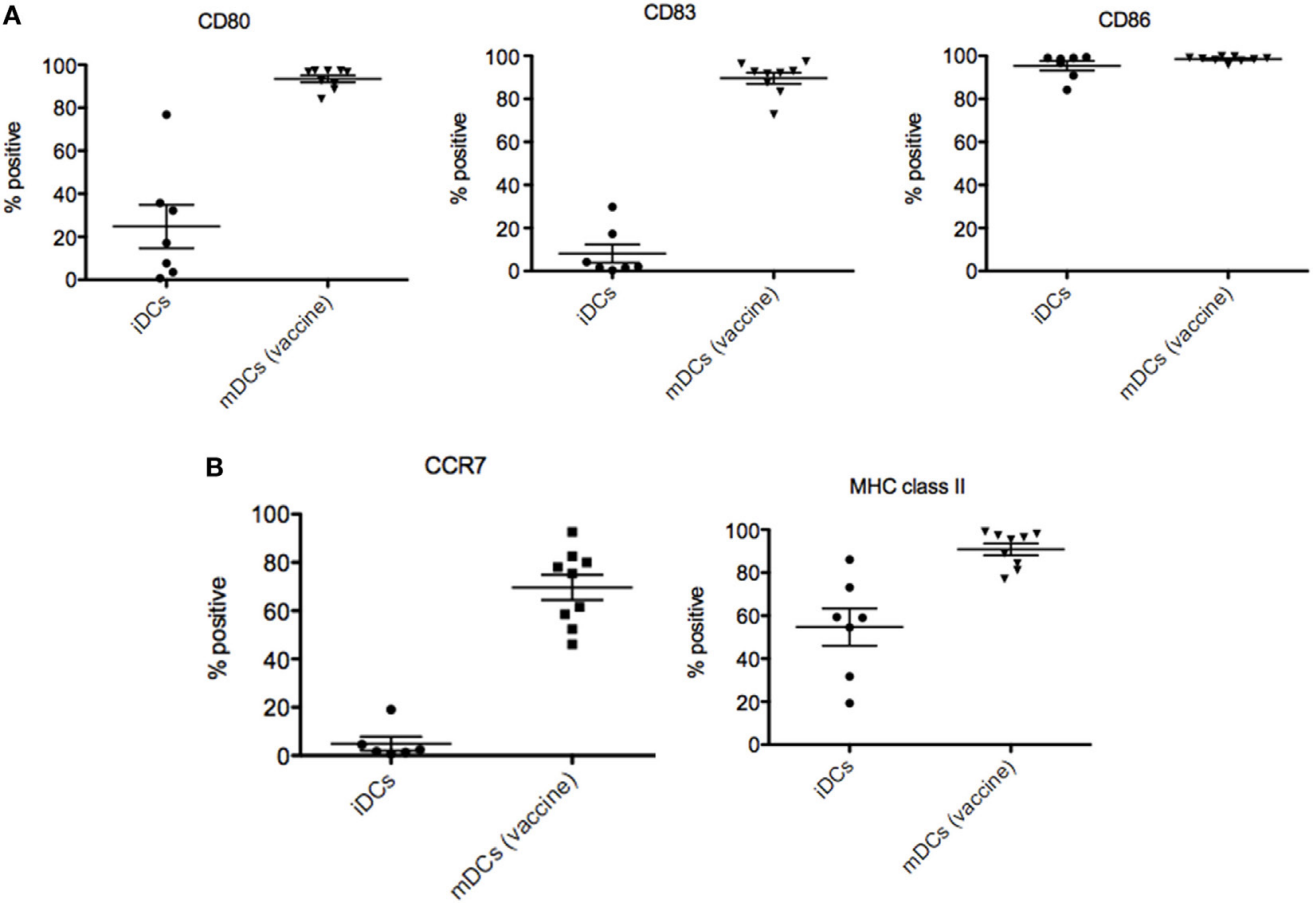
Characterization of dendritic cells (DCs) vaccines. **(A)** Increased expression of CD80, CD83, MHC class II, and CCR7 molecules on DCs surface membrane of autologous dendritic cell (ADC) compared to immature DC used as negative control. **(B)** Increased intensity of molecules (MFI) of ADC after stimulation compared to immature DC used as negative control.

Autologous dendritic cell vaccines were fabricated with no complications in all nine patients included in the study. As represented in Figure 3A, ADCV production fulfilled the release criteria showing an increased expression of costimulatory (CD80), maturation (CD83), antigen presenting [major histocompatibility complex (MHC) class II], and cytokine receptor (CCR7) molecules on DCs surface membrane when compared to immature DCs phenotype. Interestingly, not only percentage, but also the intensity of molecules (MFI) (Figure 3B) was increased in the vaccine product. Routinely, the viability of ADC was up to 80% and purity above 90%.

### Immunologic Response in Peripheral Blood

In order to monitor the effect of ADCVs administration in patients included in the trial, induction of antigen-specific T-cells was studied by a proliferation assay based on CFSE-dilution, comparing non-specific (against KLH) and specific response to ATCL. Patients treated with ADCVs increased KLH-specific and ATCL specific T-cell response detected by T-cell proliferation (CFSE-dilution) (Table 3). It is important to highlight the recognition of ATCL in eight of nine patients at some point during ADCV therapy administration. The induction of KLH-specific response in the majority of the patients indicates the potency of ADCV to generate an immune response in the patients. Of note, as shown in the same table, case DIPG-DC003 showed pre-vaccination reactivity. This could indicate spontaneous self-vaccination against tumor antigens in the case of the tumor lysate as stimuli, while the lowest reactivity against KLH (never present in non-vaccinated individuals) suggests non-specific intercurrent stimuli (i.e., infection) that could induce a basal reactivity.

**Table 3:** Percentage (%) of proliferating T-cells from PBMCs labeled with 5,6-carboxyfluorescein diacetate succinimidyl ester-dilution in response to tumor cell line lysate or KLH after substraction of signal in unstimulated control.

	Pre-vaccination		Post-1 induction		Post-2 induction		Post-3 induction		Post-4 induction	
	Tumor lysate	KLH	Tumor lysate	KLH	Tumor lysate	KLH	Tumor lysate	KLH	Tumor lysate	KLH
DIPG-DC001	–	–	–	–	3.2%	3.5%	–	–	–	–
DIPG-DC002	–	–	4%	–	–	1.4%	–	3.6%	–	–
DIPG-DC003	33%^a^	3.5%^a^	–	26%	–	16.1%	–	3.7%	3.4%	14.7%
DIPG-DC004	–	–	–	7.7%	7.1%	57.8%	2.4%	2.9%	3.6%	7.6%
DIPG-DC006	–	–	–	–	8.3%	11.6%	2.6%	1.2%	9.6%	42%
DIPG-DC007	–	–	–	–	–	–	–	17	3.3	9.3
DIPG-DC008	–	–	–	20%	nd	nd	–	–	–	–
DIPG-DC009	–	–	–	18.6%	–	6.3%	31.7%	33%	–	18.3%
DIPG-DC010	–	–	–	1.5%	–	1.5%	–	8.8%	4.3%	26.6%

*nd, not determined; (–), no proliferation*.

*^a^This pre-vaccination reactivity could indicate spontaneous self-vaccination against tumor in the case of lysate as stimuli, while the lowest reactivity against KLH (never present in non-vaccinated individuals) suggests non-specific intercurrent stimuli (i.e., infection) that could induce a basal reactivity*.

### Immunologic Response in the CSF

**Figure 4:**
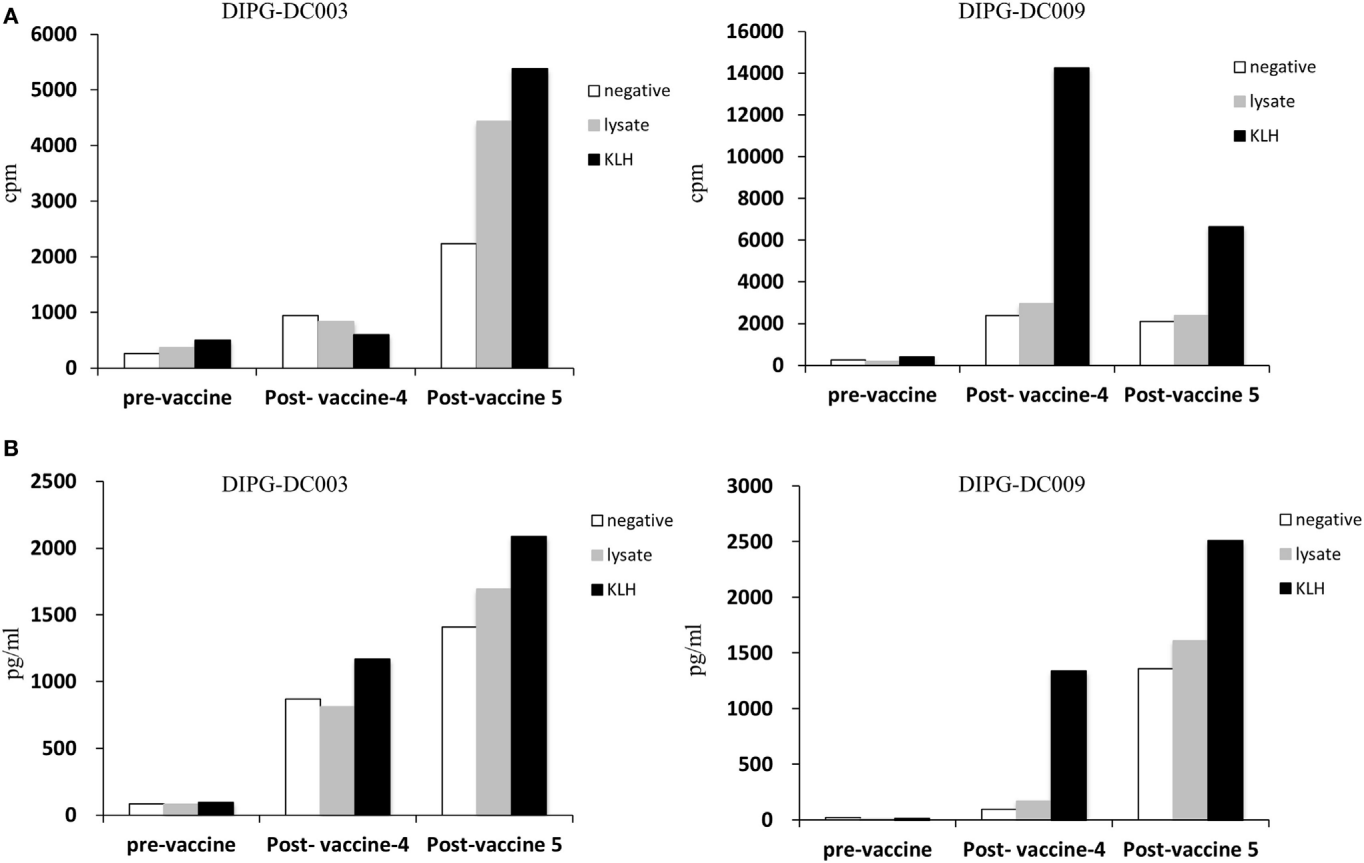
Isolated and expanded T-cell obtained from CFS. **(A)** Antigen specificity of T cell from CSF was evaluated by cell proliferation (cpm indicates thymidine incorporation; cpm = counts per million) in response to KLH or tumor cell lines lysate. **(B)** T cell activation was measured by cytokine production (IFN-γ) in response to KLH or tumor cell lines lysate compared to negative.

With the intention of identifying a closer and more suitable surrogate marker for the immune response generated by vaccination in the tumor environment, we collected CSF by serial spinal taps from the patients included in the study. If identified, T-cells were expanded *in vitro* after CD3–CD28 stimulation in the presence of rhIL-2. We were able to expand and analyze the specificity of response from T-cells isolated from two patients (DIPG-DC003 and DIPG-DC009). Interestingly, after ADCV administration, KLH responding T-cells in the CSF were detected in both patients measured by cell proliferation (Figure 4A) IFN-γ production (Figure 4B). For DIPG-DC003, ATCL specific T-cells were also identified in the CSF collected 2 weeks after the fifth ADCV dose administration showing specific anti-DIPG response.

## Discussion

This is, to our knowledge, the first clinical report of ADCVs administration using a DIPG ATCL to pulse the ADCs, after upfront RT in a homogeneous cohort of patients with newly diagnosed DIPG. Our findings show feasibility, reasonable safety of this therapeutic approach, as well as evidence of non-specific and specific antitumor immunological response generated in treated patients.

With standard therapy, the mean overall survival (OS) reported in DIPG is approximately 8–11 months, and the majority of patients succumb to disease within the first 2 years after diagnosis. Focal irradiation at diagnosis and re-irradiation at progression benefit a proportion of patients, but life expectancy is overall poor (20, 21). DIPG molecular characterization has shown unique abnormalities when compared with other non-midline pediatric high-grade glioma (HGG) and to adult HGG. Thus, we understand now DIPG not as a single entity, but as a heterogeneous group of tumors that develop in the ventral pons of school-aged children and harbors specific genetic (i.e., TP53, PDGFRA, ACVR1, and FGFR1) and more importantly epigenetic (i.e., K27M H3.3 and H3.1 variants) alterations (22). This molecular variability may explain different tumor behavior and response to RT and will be paramount for the design of future clinical trials. However, this new bulk of knowledge has not impacted significantly the OS. Additionally, current strategies including focal RT or newer delivery systems (e.g., convection-enhanced delivery) target the bulk of tumor burden. However, it has been well recognized that tumor migration and dissemination occurs early during DIPG course (15). Therefore, potentially effective strategies should aim to reach not only the macroscopically infiltrated pons but to identify and exterminate all malignant cells within the CNS.

Immunotherapy has recently gained popularity in a number of refractory adult solid tumors. In terms of effectiveness, CNS tumors disease control lags behind for a number of reasons. However, more and more IT seems to be a promising part of the armamentarium necessary to control and eradicate invasive gliomas (13). Therapeutic vaccination relies on the patient’s immune system to react to an injected tumor vaccine. ADCs are a subset of white blood cell, and play a key component to most aspects of adaptive immunity due to their central role as specialized antigen-presenting cells in the initiation and propagation phase of T-cell responses (18). Typically DCs reside as inactivated and immature state in organs and tissues at the interface of potential pathogen entry sites. After a recognized stimulus, they upregulate a cascade of events involving mostly chemokine receptors, which guide them to draining lymph nodes. There, the already mature DCs are capable of generating primary T-cell responses due to their high levels of MHC, adhesion and costimulatory molecule expression. Of note, DCs are able to present and cross-present the antigenic peptides in the context of both MHC Class II and Class I molecules, respectively, promoting CD4+ T and CD8+ cells, both fundamental for an effective cell-mediated immune response. DCs are also strong activators of natural killer and NKT cells, linking effectively the innate and adaptive immune responses. Therefore, both tumor cells with and without expression of MHC molecules can theoretically be eradicated (17, 23). HGGs have been shown to express an impressive collection of glioma-associated antigens (GAAs). Targeting specific or groups of GAAs using selected peptides (24) would inherently lead to immune escape because of the positive clonal selection of antigen-loss variants: those tumor cell clones that do not express the particular, targeted GAA or the clones that go through immune-editing and loose the expression of the antigen, will potentially escape from the immune rejection generated by the vaccination and thus have an important proliferation advantage and selection as compared to the cell clones that do express the targeted GAA. This is the main reason to use whole ATCL of different cell lines as a source of GAAs to load DCs. Our expectation was to generate a vast immune response against the most immunogenic and common DIPG antigens present in the ATCL. Vaccines generation was successfully achieved in all patients included in the trial. Our ADCV therapy was overall well tolerated and no remarkable clinical or laboratory toxicity was identified. The administration of dosages was ambulatory, offering an optimal quality of life for patients included in the trial with limited ambulatory visits or admission for inpatient therapy. Of note, patient DIPG006 developed an intratumoral hemorrhage after the second dose of ADC which prompted admission to the pediatric intensive care unit for respiratory support due to decrease in the level of consciousness. Due to neurologic deterioration, systemic steroids were administered for 3 weeks and the patient rapidly improved after a few days. It is unlikely that this hemorrhage was secondary to the ADCV, since it occurred after dose 2 when no antitumor response was identified in PBMCs. Additionally; there have been reports of spontaneous tumoral bleeds throughout DIPG disease course. Interestingly, this patient developed a strong vaccine immune response with no apparent new neurologic symptoms.

Our results show that vaccines preparation was feasible in all patients and doses administrations were well tolerated. Non-specific and antitumor lysate specific cellular response was obtained in PBMCs in the majority of patients included in the trial during therapy. We could identify a similar response in 2/9 of patients’ CSF, one of them showing specific anti-DIPG response. We speculate we could only identify two cases of response in the CSF given the low number of lymphocytes in this compartment. In summary, this ongoing trial demonstrates the feasibility of specific ADCV preparation, acceptable safety and promising preliminary specific antitumor immunoreactivity in response to ADCV. Clinical and radiologic correlation will be reported when all data are available. Additionally, it is very interesting the identification of HLA-A2-restricted immunogenic peptide derived from the K27M H3.3 mutation (25). The recognition by CTLs from patients immunized opens the possibility to specifically target the cells harboring the mutation. Nonetheless, there is clearly room for improvement for approaching DIPG with IT strategies. The immunological responses shown in our study can be surely substantiated by incorporating strategies to prevent immune resistance or editing/escape, such as the concomitant administration of immune checkpoint inhibitors that can potentiate and maintain the immune response generated by the ADCV.

## Author Contributions

MJ, DB-R, JM, OC, AMC and AMLM designed the study. PP and AG procured the tumor specimens for the cell lines, and CdT and NS characterized molecularly the samples. SP and ACM established the cell lines and tumor lysate. RC, GF-G, EA G-N and DB-R prepared the vaccines. MM, OC, VSM, and AMLM obtained clinical and laboratory data. RC, GF-G, EA G-N and DB-R analyzed immunologic data. MJ, DB-R, OC, JM, ACM and AMLM prepared the original manuscript. All authors provided manuscript review and approval.

## Conflict of Interest Statement

The authors declare that the research was conducted in the absence of any commercial or financial relationships that could be construed as a potential conflict of interest.

## Abbreviations

AEMPS, Agencia Española del Medicamento y Productos Sanitarios (Spanish regulatory agency for ATM); *ACVR1*, activin A receptor, type I; ATCL, allogeneic tumor cell-lines lysate; ADC, autologous dendritic cell; ADCV, autologous dendritic cell vaccines; AE, adverse event; APC, antigen-presenting cells; BBB, blood–brain barrier; CED, convection-enhanced delivery; CFSE, 5,6-carboxyfluorescein diacetate succinimidyl ester; CNS, central nervous system; CSF, cerebrospinal fluid; CT, computed tomography; CTL, cytotoxic T lymphocytes; DC, dendritic cells; DIPG, diffuse intrinsic pontine glioma; DNFB, dinitrofluorobenzene; EFS, event-free survival; GAAs, glioma-associated antigens; GM-CSF, granulocyte macrophage colonies stimulating factor; GMP, good manufacturing practices; GBM, glioblastoma multiforme; HGGs, high-grade gliomas; IT, immunotherapy; MHC, major histocompatibility complex; NK, natural killer; NKT, natural killer/T-cells; PBS, phosphate-buffered saline; PICU, pediatric intensive care unit; OS, overall survival; PBMC, peripheral blood mononuclear cells; *PDGFRA*, platelet-derived growth factor receptor α; RT, radiation therapy; WHO, World Health Organization; WBC, white blood cells.
