# Corrigendum: Immune Response Generated With the Administration of Autologous Dendritic Cells Pulsed With an Allogenic Tumoral Cell-Lines Lysate in Patients With Newly Diagnosed Diffuse Intrinsic Pontine Glioma

**DOI:** 10.3389/fonc.2018.00201

**Published:** 2018-06-05

**Authors:** Daniel Benitez-Ribas, Raquel Cabezón, Georgina Flórez-Grau, Mari Carmen Molero, Patricia Puerta, Antonio Guillen, E. Azucena González-Navarro, Sonia Paco, Angel M. Carcaboso, Vicente Santa-Maria Lopez, Ofelia Cruz, Carmen de Torres, Noelia Salvador, Manel Juan, Jaume Mora, Andres Morales La Madrid

**Affiliations:** ^1^Department of Immunology, Hospital Clinic, August Pi i Sunyer Biomedical Research Institute (IDIBAPS), University of Barcelona, Barcelona, Spain; ^2^Department of Clinical Trials, Hospital Sant Joan de Déu, Barcelona, Spain; ^3^Department of Neurosurgery, Hospital Sant Joan de Déu, Barcelona, Spain; ^4^Laboratory of Developmental Cancer, Institut de Recerca Sant Joan de Déu, Barcelona, Spain; ^5^Department of Oncology and Hematology, Hospital Sant Joan de Déu, Barcelona, Spain; ^6^Department of Neuro-Oncology, Hospital Sant Joan de Déu, Barcelona, Spain; ^7^Department of Immunotherapy, Hospital Sant Joan de Déu, Barcelona, Spain

**Keywords:** immunotherapy, dendritic, cell, vaccination, diffuse intrinsic pontine glioma

E. Azucena González-Navarro was not included as an author in the published article. The correct author contributions statement appears below:

## Author Contributions

MJ, DB-R, JM, OC, AMC and AMLM designed the study. PP and AG procured the tumor specimens for the cell lines, and CdT and NS characterized molecularly the samples. SP and ACM established the cell lines and tumor lysate. RC, GF-G, EA G-N and DB-R prepared the vaccines. MM, OC, VSM, and AMLM obtained clinical and laboratory data. RC, GF-G, EA G-N and DB-R analyzed immunologic data. MJ, DB-R, OC, JM, ACM and AMLM prepared the original manuscript. All authors provided manuscript review and approval.

The authors apologize for this error and state that this does not change the scientific conclusions of the article in any way.

The original article has been updated.

## Conflict of Interest Statement

The authors declare that the research was conducted in the absence of any commercial or financial relationships that could be construed as a potential conflict of interest.

